# Change detection in audio-visual space: neural evidence for age effects

**DOI:** 10.3389/fnagi.2026.1860627

**Published:** 2026-08-11

**Authors:** Benjamin Stodt, Daniel Neudek, Rainer Martin, Edmund Wascher, Stephan Getzmann

**Affiliations:** 1 Leibniz Research Centre for Working Environment and Human Factors at the TU Dortmund (IfADo), Dortmund, Germany; 2 Institute of Communication Acoustics, Ruhr-Universität Bochum, Bochum, Germany

**Keywords:** aging, change detection, event-related potentials, multisensory integration, spatial perception

## Abstract

**Introduction:**

The ability to perceive spatial changes is crucial for everyday life. However, the effects of age on multisensory integration in spatial change detection are still less researched. Here, we investigated whether audio-visual stimulation enhances spatial change detection compared to auditory-only processing, and whether older adults benefit more from multisensory stimulation than younger adults.

**Methods:**

In an active oddball task, younger and older participants had to detect spatial changes in azimuth (in the horizontal plane) or distance (in the depth dimension). Stimuli were presented as auditory-only or audio-visual (spatially congruent). Behavioral performance and event-related potentials indexing sensory processing (P1, N1, P2), automatic change detection (mismatch negativity), attention orienting (P3a), and evaluation (P3b) were measured.

**Results:**

Detection accuracy was higher for azimuth than distance changes in auditory-only conditions, but this difference was reduced with audio-visual stimulation. Older adults showed comparable behavioral performance to younger adults across both conditions, with no additional benefit from multisensory input. However, older adults exhibited enhanced early sensory processing (larger N1, P2 amplitudes), reduced MMN differentiation between azimuth and distance deviations, and later evaluative processing that was primarily modulated by spatial dimension and stimulation modality. Thus, age-related neural differences were component-specific and depended on spatial dimension and stimulation modality.

**Discussion:**

Taken together, audio-visual integration facilitated spatial change detection, particularly in the depth dimension. While older adults maintained behavioral performance possibly through compensatory neural mechanisms involving enhanced attentional allocation, the use of multisensory stimuli for spatial change detection appears to be largely preserved in aging.

## 1 Introduction

The perception of changes in the environment is of vital importance for our everyday life. This applies, for example, to safe behavior in traffic, which depends on quickly perceiving approaching vehicles or changing traffic lights. To respond to such an event adequately, it must in principle be perceptible. Thus, a change in the visual environment must occur within our field of view, whereas a change in the acoustic environment must not be masked by background noise. However, even easily visible and audible changes are not perceptible per se, as impressively demonstrated by examples of change blindness (e.g., Gibbs et al., 2016), or its auditory analog of change deafness (Gaston et al., 2017). These examples show that our ability to perceive changes in space closely depends on the interaction of various sensory and cognitive functions.

The cognitive mechanisms of change perception can be experimentally operationalized by using oddball paradigms. In a typical active oddball paradigm, a sequence of stimuli is presented consisting of frequent “standards” and rare “targets.” Standard and target stimuli differ in certain features, such as frequency, length, or spatial position, and the participant’s task is to respond as quickly as possible to the target and any such changes. The cognitive subprocesses of this change detection can be revealed by analysis of event-related potentials (ERPs), which are time-locked responses extracted from the continuous electroencephalogram (EEG) and reflect successive processing stages: The P1 component predominantly reflects the encoding of physical stimulus properties and is largely sensory, with minimal influence from cognitive or attentional factors (Eggermont and Ponton, 2002; Grunwald et al., 2003) while the N1 marks the onset of attentional orientation to novel or deviant stimuli or events (Näätänen and Picton, 1987). Subsequently, the P2 is linked to higher-level operations such as stimulus classification and relevance evaluation (Arnott et al., 2011). Oddball paradigms reliably elicit a systematic pattern of deviance-related ERPs derived by subtracting responses to standards from those to targets, most prominently the mismatch negativity (MMN) and the P3 complex (Korka et al., 2022; Polich, 2007). The MMN indexes automatic detection of change (Näätänen et al., 2007, 2012; Rutiku et al., 2024), for example triggered by a switch in spatial location (Deouell et al., 2003, 2006; Deouell and Bentin, 1998; Lewald et al., 2018; Paavilainen et al., 1989; Schröger and Wolff, 1997; Sonnadara et al., 2006b,2006a), while the P3a reflects the subsequent orienting of attention toward unexpected stimuli (Friedman et al., 2001; Sanada et al., 2025). Within the predictive coding framework, both components indicate violations of the brain’s predictions, with MMN representing lower-level and P3a higher-level prediction errors (Lecaignard et al., 2015). Both often occur sequentially, and their amplitudes scale with stimulus salience and unexpectedness, though they can be dissociated depending on stimulus features (Coy et al., 2024; Horváth et al., 2008). Finally, the parietal P3b reflects later stimulus evaluation, context updating, and allocation of cognitive resources, with amplitude and latency modulated by target discriminability and mental workload (Kok, 2001; Lewald et al., 2018; Polich, 2007; Verleger et al., 2005; Wronka et al., 2012).

The sensory and cognitive processes involved in change perception undergo alterations with age. Sensory impairments, particularly in vision and hearing, are common in older adults (Xiao et al., 2021), and there is also evidence for heterogeneous age-related changes in cognitive functions during healthy aging (Prince et al., 2024). However, research on age effects on spatial cognition and change detection revealed mixed findings (Klencklen et al., 2012). Some studies showed a decline in the ability to detect changes in the visual environment with age (Tran et al., 2021), possibly due to reduced processing speed (Costello et al., 2010). With respect to hearing, aging has an impact on peripheral as well as on early (primary) and higher-order (posterior-dorsal and dorsolateral prefrontal) auditory regions, which contribute to the cortical representation of sound location (van der Heijden et al., 2019). This can result in a decline in temporal processing, encoding of monaural and binaural spatial cues, and thus sound localization (Dobreva et al., 2011; Eddins et al., 2018; Freigang et al., 2015; Freigang et al., 2014). There is also evidence that aging is associated with reduced sensitivity to abrupt changes in complex acoustic scenes, while the ability to detect changes in predictable structures is preserved (de Kerangal et al., 2020).

Age influences on change perception in auditory space were also observed in ERP studies. Focusing on the location-evoked acoustic change complex (ACC), an ERP pattern associated with a spatial change of continuous sound stimulus (Fan et al., 2022), a reduction of the N1-P2 complex evoked by switches in the horizontal plane was observed in older compared to younger adults (Wang et al., 2024), while the dependency of ACC on the angle shifts was preserved (Nie et al., 2024). In addition, age-related changes in MMN have been observed (e.g., Cheng et al., 2013; Chow et al., 2025; Cooper et al., 2006; Getzmann et al., 2024; Ruzzoli et al., 2012), suggesting that the automatic processing of changes in spatial position is impaired in older adults (Freigang et al., 2014). This was true especially for switches at peripheral locations, while change detection was largely preserved for central locations. It was suggested that older adults more actively engage top-down attentional processes to detect small spatial changes in the peripheral acoustic field, in line with the assumption of compensatory mechanisms in higher age, as often indicated by an increased N1 amplitude (Tomé et al., 2015).

Stronger N1 amplitudes in older adults were also observed in a recent spatial change detection study (Stodt et al., 2025). Here, an oddball paradigm was used with switches of sound sources both in azimuth and distance, revealing a reduced MMN and a delayed P3b in older adults, but no differences in detection accuracy. There were also no age differences observed for switches in azimuth and distance, which is noteworthy given that the mechanisms of sound localization differ profoundly in the horizontal and sagittal plane. While in the horizontal plane, binaural cues like interaural level differences (ILD) and interaural time differences (ITD) are crucial for accurate localization, for distance perception in the sagittal plane sound intensity plays a major role, decreasing as distance increases (Kolarik et al., 2016; Zahorik et al., 2005). However, this is most effective when the source remains constant and the listener is familiar with the environment. For unfamiliar sounds or varying intensities, additional cues like the direct-to-reverberant energy ratio (DRR) and changes in spectral characteristics contribute to distance perception (Kopčo and Shinn-Cunningham, 2011; Spiousas et al., 2017). Auditory distance perception thus relies on more subtle cues, making it less precise, especially for nearby sources (Arend et al., 2021), which often leads to an underestimation of proximity (Andreeva et al., 2024). In the auditory oddball task, switches in distance elicited smaller and delayed P3b responses compared to azimuth changes (Stodt et al., 2025). This pattern may reflect lower salience of distance cues, but could also result from their reduced reliability and discriminability, as distance perception relies on more variable and context-dependent acoustic information than azimuth perception.

While such previous research has established important findings for auditory space perception, the use of auditory-only paradigms potentially overlooks the multimodal nature of real-world perception, in which environmental changes are often perceived through bimodal sensory input. Research demonstrates that the integration of both auditory and visual modalities typically leads to substantially improved spatial perception (such as localization accuracy, speed, and confidence), since the brain combines the strengths of both modalities, reducing uncertainty and exploiting complementary spatial information (e.g., Teder-Sälejärvi et al., 2005). For example, by incorporating visual references into auditory tasks (such as cues related to room size and depth), the visual context significantly enhances auditory processing efficiency and accuracy (Van De Par et al., 2022). Thus, there is evidence that distance perception is improved by congruent visual stimuli in audio-visual reverberant environments (Anderson and Zahorik, 2014; Calcagno et al., 2012; Zahorik and Wightman, 2001). Furthermore, multimodal approaches are particularly valuable for exploring how auditory and visual systems interact in contexts demanding heightened attention (Breuer et al., 2022; Hohmann et al., 2020), as visual objects enhance the overall impression and intensify presence by directing attention (Best et al., 2007). Finally, despite age-related changes in auditory and visual localization, the interactions between both modalities did not differ between younger and older individuals (Xiong et al., 2022).

The present study investigated whether spatial change detection in azimuth and distance benefits from audio-visual perception over auditory-only processing, with a specific focus on differences related to age. We extended our oddball paradigm (Stodt et al., 2025) to include congruent audio-visual stimuli, allowing a direct contrast between unimodal auditory and bimodal audio-visual change detection. We therefore compared data on auditory change detection recently published in Stodt et al. (2025) with data on spatial change detection of congruent audio-visual stimuli. At the behavioral level, it was expected that older participants would show a greater benefit from audio-visual stimulation than younger participants due to age-related changes in auditory processing, and that this benefit would be more pronounced in terms of distance than in the horizontal plane due to differences in auditory spatial perception. At the neurocognitive level, it was expected that age differences would be more evident in the later processes of decision-making and response selection (indicated by P3b) than in the earlier processes of change detection and attention focusing (indicated by MMN and P3a).

## 2 Materials and methods

### 2.1 Participants

**FIGURE 1:**
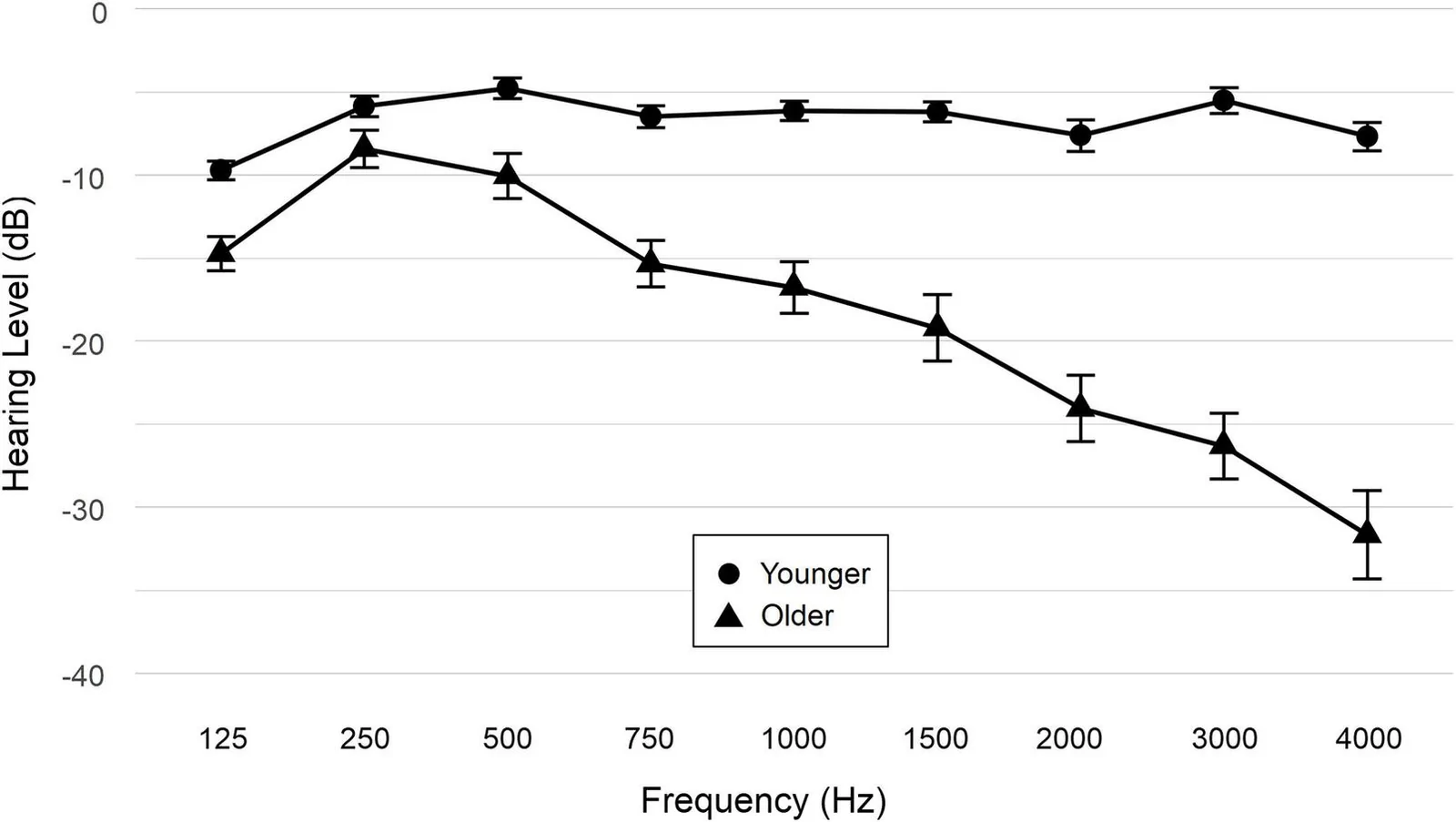
Pure-tone audiograms averaged across younger and older participants. Error bars represent standard deviations.

Data were collected between May 2023 and March 2024 in Germany. The experiment involved 22 younger (mean age 24.00 years, SD = 3.19, range 20–31; 10 female, 12 male) and 22 older (mean age 64.77 years, SD = 4.47, range 55–70; 13 female, 9 male) participants. All participants were right-handed and reported having no serious medical conditions or taking any medication that might affect their cognitive performance during the experiment. All participants self-reported normal vision and hearing. Additionally, participants performed pure-tone standard audiometry using frequencies of 125, 250, 500, 750, 1,000, 1,500, 2,000, 3000, and 4,000 Hz (Oscilla USB100, Inmedico, Lystrup, Denmark), indicating hearing thresholds of both age groups within an acceptable range (< 35 dB hearing loss at frequencies ≤ 4,000 Hz). The older group had overall higher hearing thresholds than the younger group, especially at higher frequencies (see Figure 1). Accordingly, a repeated-measures ANOVA indicated main effects of age, *F*_1,42_ = 34.00, *p* < 0.001, η_*p*_^2^ = 0.447, and frequency, *F*_1,42_ = 20.49, *p* < 0.001, η_*p*_^2^ = .328, as well as an interaction of age and frequency, *F*_8,336_ = 16.26, *p* < 0.001, η_*p*_^2^ = 0.279.

A screening for cognitive impairment (Montreal Cognitive Assessment, MoCA; Nasreddine et al., 2005) was administered only to the older participants and revealed no indication of moderate or severe cognitive decline in this age group (*M* = 26.15, *SD* = 2.48). The participants were provided with written task instructions and were paid 12€ per hour or received equivalent study credits. All participants provided written informed consent prior to the study. The study conformed to the Code of Ethics of the World Medical Association (Declaration of Helsinki) and was approved by the local Ethical Committee of the Leibniz Research Centre for Working Environment and Human Factors, Dortmund, Germany.

### 2.2 Test environment and stimuli

The experimental room (7.2 m wide × 12.0 m long × 3.4 m high; broadband reverberation time *T*_60_ ≈ 0.8 s) was the same as described in Stodt et al. (2024, 2025). Participants were positioned on a height-adjustable chair at an ear height of 1.8 m between the left and right walls, 2 m from the back wall. A chin rest was used to ensure a frontal gaze direction. Five wooden boxes were placed in front of the participants, three in the median plane at distances of 2, 4, and 8 m (near, center, and far, respectively), and two to the left and right of the central box (θ = ± 24°). Each box housed a loudspeaker (Visaton SC 8 N, 8 ohms, 8 cm) and a small screen (Samsung QB13R, 12.9”) for audio-visual stimulus presentation. The boxes were positioned at a height of 1.14 m, measured from the floor to the center of the loudspeaker.

The auditory-only stimulus consisted of a 500-ms pink noise burst (10-ms fade-in, 10-ms fade-out, band-pass filtered to a frequency range of 125 Hz to 4 kHz), while the audio-visual stimulus consisted of the simultaneous presentation of this auditory stimulus and a gray full-screen visual stimulus (hex code #d3d3d3, 4 frames fade-in, 4 frames fade-out) at the same spatial location as the auditory stimulus. In order to ensure precise timing of sound arrival at the participants’ ears regardless of the position of the loudspeakers, the time-of-arrival of the auditory stimulus was measured and compensated for.

### 2.3 Experimental paradigm and procedure

In the spatial change detection (oddball) paradigm, participants were presented with sequences of either auditory-only or audio-visual stimuli. The inter-stimulus interval (ISI) was uniformly sampled between 1,300 and 1,700 ms with 1-ms resolution, yielding an average ISI of 1,500 ms across trials. Each condition comprised 900 trials, presented in separate blocks, with the order of auditory-only and audio-visual blocks counterbalanced across participants. Short breaks were provided after every 300 trials. In 684 of the trials, the stimuli were emitted from the center position (76% standard trials), and in the remaining trials, they were emitted randomly from the near, far, left, or right position (54 trials each, 6% target trials). It was ensured that a target was followed by between two and six standards. The participants’ task was to indicate the position of the target when it appeared. Responses were given using a two-axis joystick: leftward movements corresponded to left targets and rightward movements to right targets, while pulling the joystick toward oneself indicated near targets and pushing it away from oneself indicated far targets. No response was required when the standard stimulus (center loudspeaker) was presented.

### 2.4 EEG recording and preprocessing

EEG was recorded using 64 Ag/AgCl electrodes (EasyCap, Wörthsee, Germany) based on the 10–20 system and a QuickAmp DC Amplifier (BrainVision, Gilching, Germany), with AFz serving as ground electrode and FCz as reference electrode. The sampling rate was 1,000 Hz, electrode impedances were kept below 10 kΩ.

EEG data was pre-processed offline using EEGLAB (v2025.0; Delorme and Makeig, 2004) and ERPLAB (v12.01; Lopez-Calderon and Luck, 2014) toolboxes in MATLAB. First, the raw data was filtered using a Hamming-windowed sinc FIR high-pass filter (cutoff 0.25 Hz, filter length 6,601, transition bandwidth 0.5 Hz) and low-pass filter (cutoff 33.75 Hz, filter length 441, transition bandwidth 7.5 Hz). EEG channels with excessive noise or flat lines were removed and interpolated using spherical interpolation. The EEG signals were then re-referenced to the average of all 64 electrodes and cleaned using independent component analysis (ICA). A subset of the EEG data was therefore used, with cut-off frequencies of 1 and 30 Hz. This subset was down-sampled to 200 Hz for faster computation with every second trial being included. The continuous EEG signal was then divided into 1,200 ms stimulus-locked epochs covering the period from −200 to 1,000 ms relative to stimulus onset (baseline −200–0 ms). Epochs in which participants did not respond correctly (i.e., false or missing responses to a target or responses to a standard) as well as each first standard following a target were excluded. Automatic artifact rejection further removed on average *M* = 24.18, *SD* = 17.13 epochs per participant (including standard and target trials). There was no difference in the number of excluded epochs between age groups for either stimulation condition (*M*_*auditory*–*only*,young_ = 22.86, *SD* = 14.52, *M*_*auditory*–*only*,old_ = 23.95, *SD* = 17.29, *t* = −0.23, *p* = 0.822; *M*_*audio*–*visual*,young_ = 23.45, *SD* = 16.32, *M*_*audio*–*visual*,old_ = 26.45, *SD* = 20.77, *t* = −0.53, *p* = 0.597). Then, the ICA was applied using the ICLabel algorithm (v1.6, Pion-Tonachini et al., 2019), and *M* = 28.82, *SD* = 6.86 components per participant were removed, which had a probability of < 30% for the brain category or > 30% for the eye category. Further, *M* = 2.51, *SD* = 8.26 remaining artifactual and noisy epochs (including standard and target trials) were removed by applying amplitude thresholds. The number of excluded epochs did not differ between age groups in either stimulation condition (*M*_*auditory*–*only*,young_ = 1.55, *SD* = 4.30, *M*_*auditory*–*only*,old_ = 2.05, *SD* = 4.46, *t* = −0.38, *p* = 0.707; *M*_*audio*–*visual*,young_ = 4.68, *SD* = 15.05, *M*_*audio*–*visual*,old_ = 1.77, *SD* = 3.31, *t* = 0.89, *p* = 0.385). Finally, the resulting data and the obtained independent component weights were transferred back to the original EEG data.

### 2.5 Behavioral analysis

The percentage of correct responses and mean response times were computed for each participant and separately for auditory-only and audio-visual stimulation. Trials with response times faster than 100 ms (i.e., premature responses) were excluded. Responses were averaged for the left and right targets (azimuth dimension) and for the near and far targets (distance dimension). Accuracy data was logit-transformed for subsequent analyses to correct for variance heterogeneity. Accuracy and response time data were submitted to repeated-measures mixed three-way ANOVAs with within-subjects factors dimension (azimuth vs. distance) and stimulation (auditory-only vs. audio-visual), and the between-subjects factor age (younger vs. older). *Post-hoc t*-tests were corrected for multiple testing using false discovery rate correction (Benjamini and Hochberg, 1995).

### 2.6 EEG analysis

Epochs containing standard trials were averaged for each participant and separately for auditory-only and audio-visual stimulation. For target trials, epochs were averaged across left and right positions (azimuth dimension) and near and far positions (distance dimension). Epochs in which the participants did not answer correctly as well as the first standard trials following a target trial were not included in the EEG analysis.

To parameterize the ERPs of interest, the waveforms were averaged across clusters of electrodes. Electrode clusters were selected a priori based on previous literature (e.g., Johnson, 1993; Näätänen et al., 2007; Näätänen and Picton, 1987; Polich, 2007) and confirmed by visual inspection of the scalp topographies in the present data (Figure 3). Averaging across neighboring electrodes increases the signal-to-noise ratio and reduces the influence of electrode-specific noise on component estimation. Amplitudes and latencies were determined using time windows derived from the literature. The P1, N1, and P2 components were analyzed at six fronto-central electrodes (Fz, F1, F2, FCz, FC1, FC2), with peak measures extracted within predefined time windows (P1: 40–70 ms, N1: 50–150 ms, P2: 100–400 ms). Early ERP components were computed from target-evoked waveforms, whereas MMN, P3a, and P3b were derived from difference waveforms obtained by subtracting ERPs elicited by standard stimuli from those elicited by target stimuli. The MMN and P3a were determined across the same six fronto-central electrodes as used for the P1-N1-P2 complex in the time range of 0–300 ms, and 150–600 ms, respectively. The P3b was determined across a centro-parietal cluster of electrodes (CP1, CPz, CP2, P1, Pz, P2) in the time range of 200–999 ms. MMN, P3a, and P3b were characterized by mean amplitudes and fractional area latencies (FAL; Kiesel et al., 2008). The use of the FAL—defined as the timepoint at which the ERP waveform reaches a certain (here 50%) percentage of its total area—has advantages in noisy and weak ERPs (Wascher et al., 2022). MMN, P3a, and P3b mean amplitudes were computed across a time window of 20 ms around the individual FAL.

ERP amplitudes and latencies were submitted to repeated-measures ANOVAs with within-subjects factors dimension (azimuth vs. distance) and stimulation (auditory-only vs. audio-visual), and the between-subjects factor age (younger vs. older). *Post-hoc t*-tests were used to clarify potential interaction effects using false discovery rate correction. Statistical analyses were carried out using R (v.4.5.1) in RStudio (v.2025.05.1; Posit Team, 2025). We used the MATLAB function from Liesefeld (2018) to implement a jackknifing procedure, which helped mitigate potential noise from individual participants’ data that could skew averaged ERP measures. This method involved calculating *n* average scores (e.g., ERP amplitude or latency) by excluding one participant at a time. The result was a set of *n* scores, each representing the grand average of *n*-1 participants (Miller et al., 2009; Smulders, 2010). The jackknife technique reduced data noise without excluding participants or compromising ERP waveforms (Wascher et al., 2022).

## 3 Results

### 3.1 Performance

**FIGURE 2:**
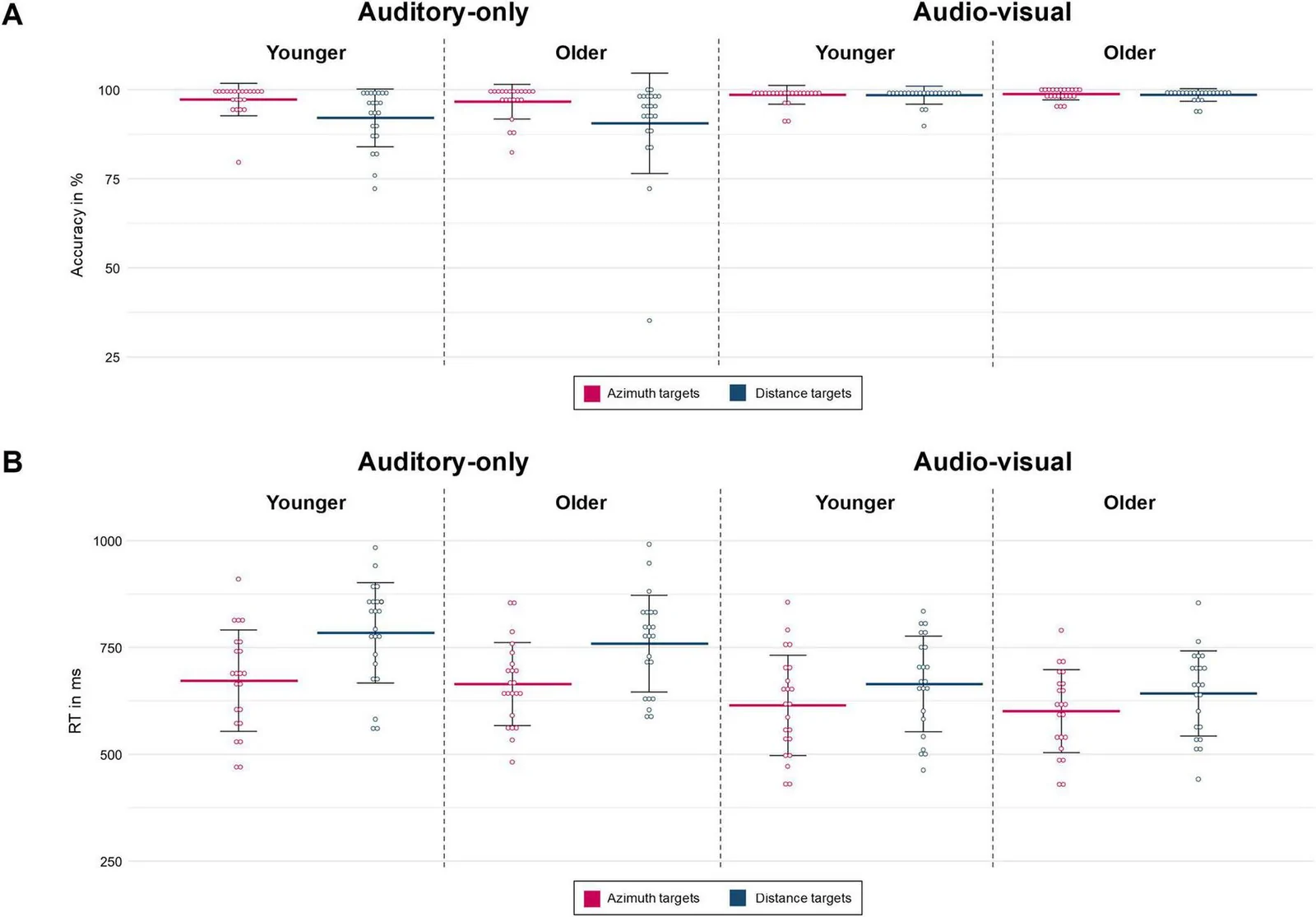
Behavioral results. Response **(A)** accuracy and **(B)** time in detecting targets in azimuth and distance, shown for auditory-only and audio-visual stimulation and both age groups. Horizontal bold lines represent the mean values over all participants, vertical bars represent ± 1 SD, and dots indicate the individual mean values.

The mean accuracy in target detection, averaged across stimulus modality, was higher in the azimuth than the distance dimension (*M*_*azimuth*_ = 97.86%, *SD* = 3.71; *M*_*distance*_ = 95.02%, *SD* = 8.92). In addition, accuracy was higher with audio-visual than auditory-only stimulation (*M*_*auditory*–*only*_ = 94.21%, SD = 9.09 vs. *M*_*audio*–*visual*_ = 98.67%, *SD* = 2.16; Table 1). The difference in accuracy between the azimuth and distance dimension was more pronounced with auditory-only than audio-visual stimulation, according to an interaction between target dimension and stimulation (Figure 2). *Post-hoc t*-tests showed a significant effect of target dimension with auditory-only stimulation (*M*_*auditory*–*only*,azimuth_ = 97.01%, *SD* = 4.64, *M*_*auditory*–*only*,distance_ = 91.41%, *SD* = 11.39, *t* = 5.42, *p* < 0.001), but not with audio-visual stimulation (*M*_*audio*–*visual*,azimuth_ = 98.72%, *SD* = 2.19, *M*_*audio*–*visual*,distance_ = 98.62%, *SD* = 2.15, *t* = 0.78, *p* = 0.442).

**TABLE 1:** Results of the 2 × 2 × 2 ANOVAs of response accuracy and response times with age, dimension, and stimulation as independent factors (F-statistics, *p*-values, and effect sizes).

Effect	Response accuracy			Response times		
	*F*	*p*	η _*p*_^2^	*F*	*p*	η _*p*_^2^
Age	0.08	0.783	0.002	0.31	0.582	0.007
Stimulation	**34.33**	**< 0.001**	**0.450**	**105.14**	**< 0.001**	**0.715**
Dimension	**26.52**	**< 0.001**	**0.387**	**173.08**	**< 0.001**	**0.805**
Age × stimulation	0.19	0.668	0.004	0.01	0.945	< 0.001
Age × dimension	0.01	0.928	< 0.001	1.32	0.258	0.003
Stimulation × dimension	**23.51**	**< 0.001**	**0.359**	**86.33**	**< 0.001**	**0.673**
Age × stimulation × dimension	< 0.01	0.989	< 0.001	0.39	0.534	0.009

Significant results are printed in bold.

The mean response times were lower in the azimuth than the distance dimension (*M*_*azimuth*_ = 638.50 ms, *SD* = 110.49 ms vs. *M*_*distance*_ = 712.86 ms, *SD* = 124.50 ms) and with audio-visual than auditory-only stimulation (*M*_*auditory*–*only*_ = 720.18 ms, *SD* = 121.90 ms vs. *M*_*audio*–*visual*_ = 631.19 ms, *SD* = 107.88 ms; see Table 1). According to the interaction effect between target dimension and stimulation, the difference in response times between azimuth and distance targets was significant with both auditory-only stimulation (*M*_*auditory*–*only*,azimuth_ = 668.55 ms, *SD* = 107.20, *M*_*auditory*–*only*,distance_ = 771.81 ms, *SD* = 114.53, *t* = −13.51, *p* < 0.001) and audio-visual stimulation (*M*_*audio*–*visual*,azimuth_ = 608.46 ms, *SD* = 106.59, *M*_*audio*–*visual*,distance_ = 653.92 ms, *SD* = 105.50, *t* = -9.09, *p* < 0.001). Although both tests showed significant differences, the difference in response times between spatial dimensions was larger in the auditory-only condition than in the audio-visual condition, reflecting a reduced benefit of azimuth targets when visual cues were present.

For both accuracy and response times, no effect of age was observed.

### 3.2 Event-related potentials

**FIGURE 3:**
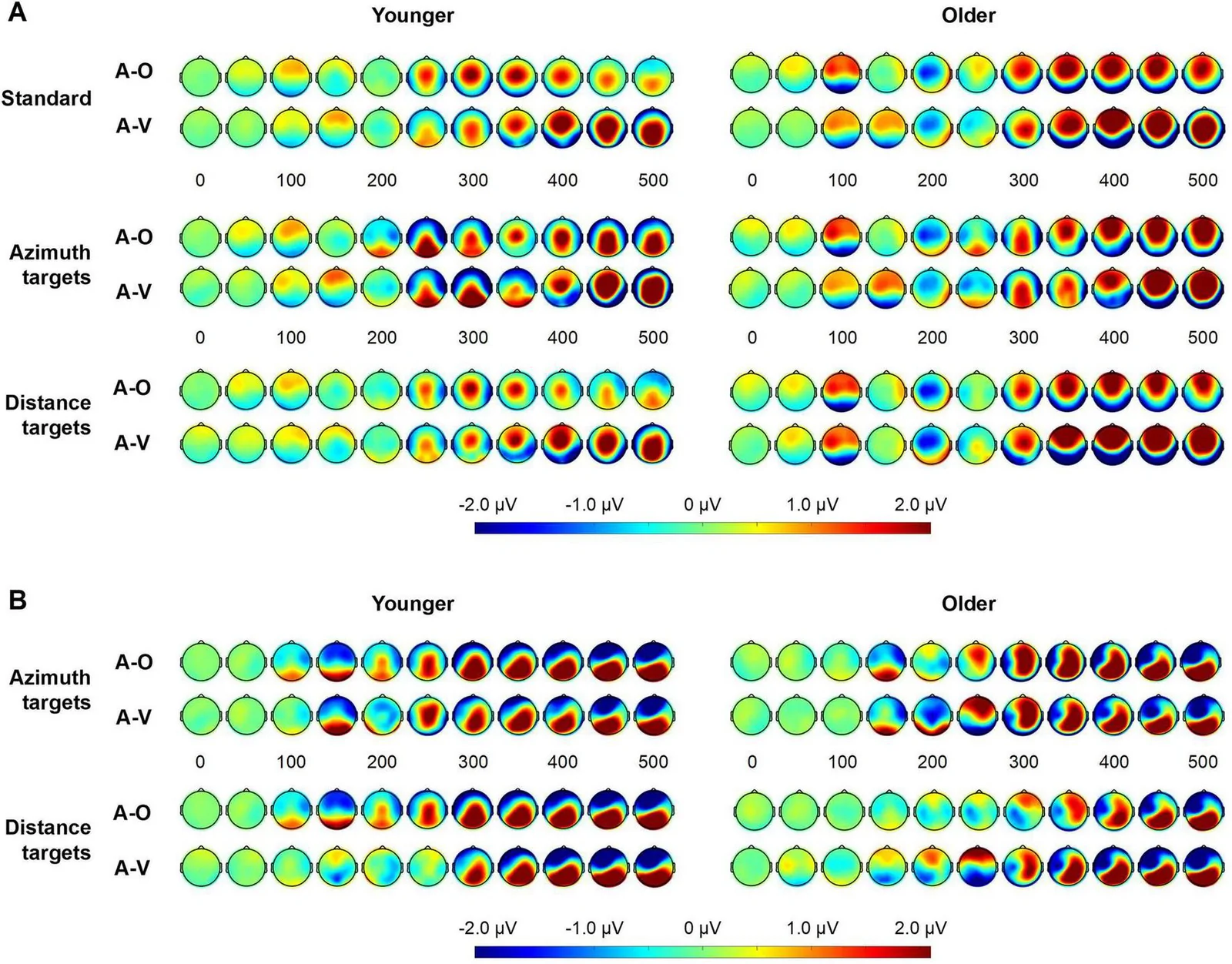
Topographies. **(A)** Averaged EEG topographies for the first 250 ms after onset of standard and target stimuli, shown separately for azimuth and distance targets and auditory-only (A–O) and audio-visual (AV) stimulation and for younger and older participants. The time ranges cover the early P1, N1, and P2 potentials. **(B)** Averaged topographies of the target-minus-standard difference waveforms for the first 500 ms after stimulus onset, covering the MMN, P3a, and P3b.

**FIGURE 4:**
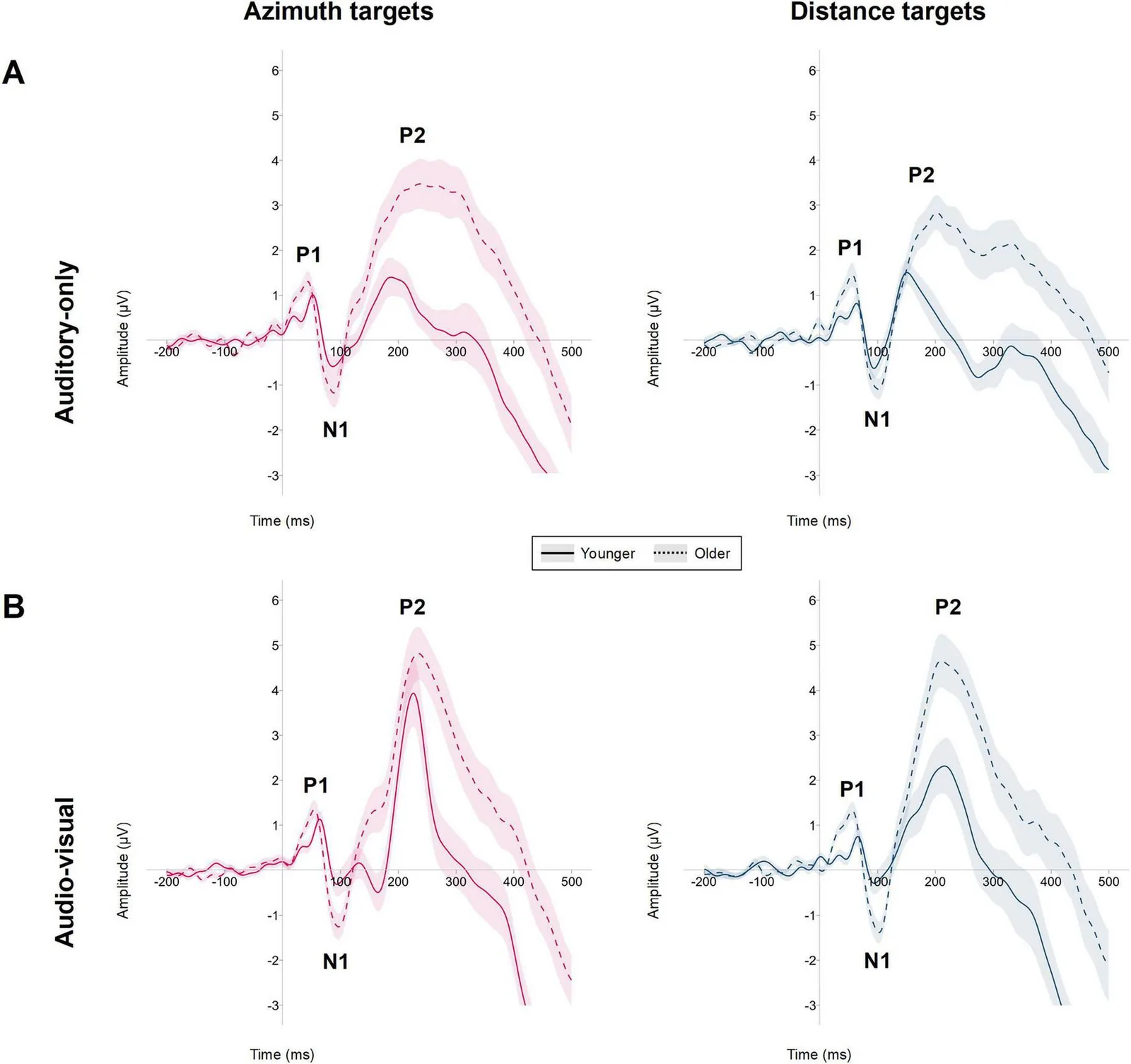
Grand averaged fronto-central ERP waveforms of target trials, shown separately for distance and azimuth targets, younger and older participants, and **(A)** auditory-only and **(B)** audio-visual stimulation. Shaded areas refer to the range of ± one standard error, the P1-N1-P2 complex is marked.

**FIGURE 5:**
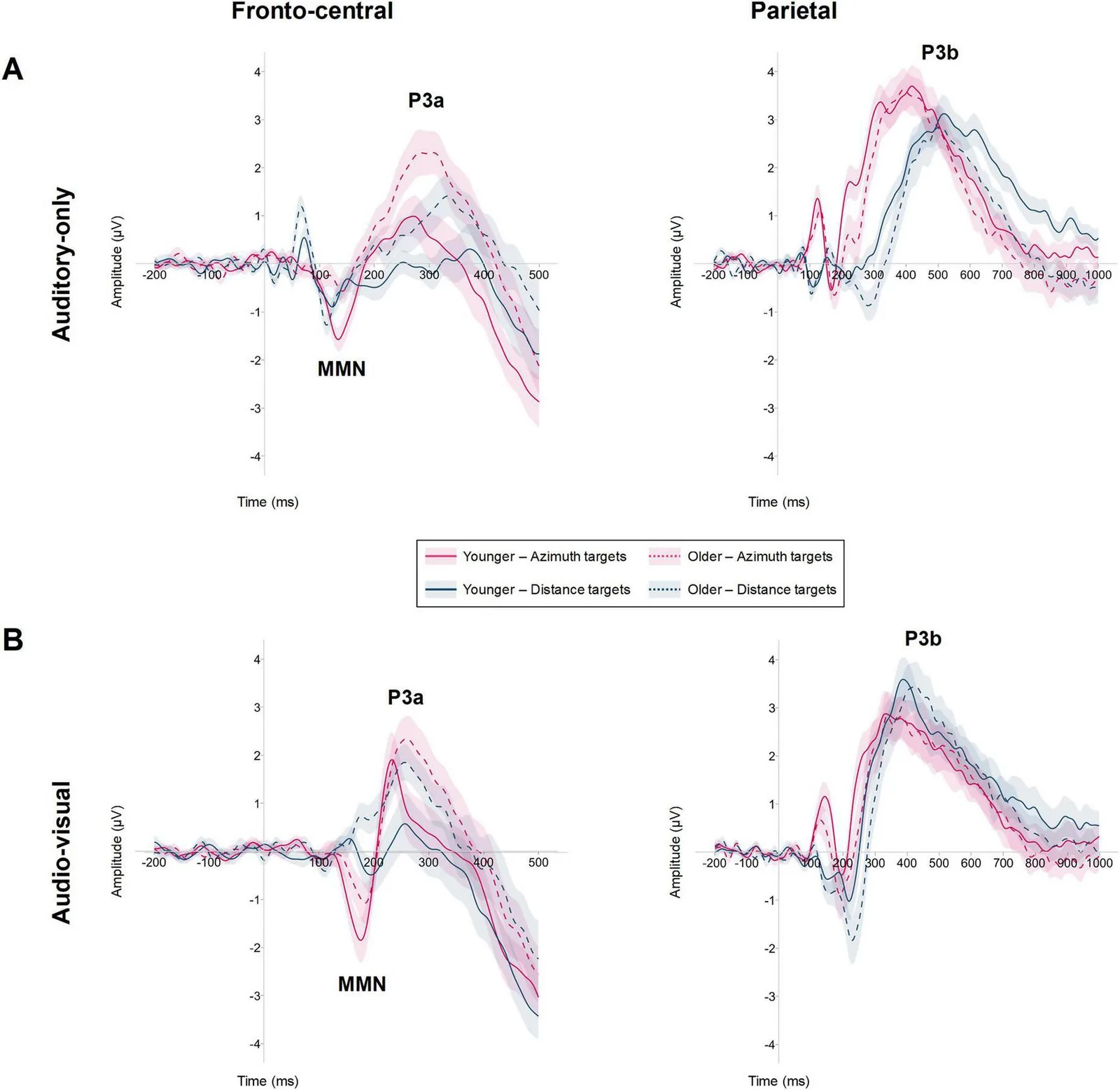
Grand averaged fronto-central and parietal ERP difference waveforms (target minus standard), shown separately for distance and azimuth targets, for younger and older participants, and for **(A)** auditory-only and **(B)** audio-visual stimulation. Shaded areas refer to the range of ± one standard error, MMN, P3a, and P3b components are marked.

In the topographies of the ERPs, a characteristic P1-N1-P2 complex appeared over fronto-central areas within the first 250 ms after stimulus onset (Figure 3A). In the target-minus-standard differential waveforms, the MMN and P3a became evident over fronto-central areas at approximately 150 ms and 250 ms, respectively, and the P3b over centro-parietal areas at 300 ms (Figure 3B). Table 2 summarizes the results of the ANOVAs examining the effects of age, stimulation, and target dimension on amplitudes and latencies of the ERPs of interest. Potential main and interaction effects are described in the following. Figures 4, 5 show the grand-averaged ERP waveforms for the early (P1, N1, P2) and later (MMN, P3a, P3b) ERP components across the different target dimensions, age groups, and stimulation modalities. Tables 3, 4 summarize amplitudes and latencies of the ERPs across age group, stimulation type, and target dimension, including their combinations.

**TABLE 2:** Results of 2 ×2 × 2 ANOVAs of ERPs with age, dimension, and stimulation as independent factors (F-statistics, *p*-values, and effect sizes).

		Amplitude (μV)			Latency (ms)		
ERP	Effect	*F*	*p*	η _*p*_^2^	*F*	*p*	η _*p*_^2^
P1	Age	2.90	0.096	0.065	**10.13**	**0.003**	**0.194**
	Stimulation	0.01	0.909	< 0.001	**29.18**	**< 0.001**	**0.410**
	Dimension	2.84	0.100	0.063	**31.79**	**< 0.001**	**0.431**
	Age × stimulation	0.22	0.644	0.005	0.20	0.656	0.005
	Age × dimension	**6.58**	**0.014**	**0.136**	0.11	0.745	0.003
	Stimulation × dimension	1.11	0.298	0.026	**17.09**	**< 0.001**	**0.289**
	Age × stimulation × dimension	0.01	0.931	< 0.001	0.16	0.694	0.004
N1	Age	**5.47**	**0.024**	**0.115**	1.48	0.231	0.034
	Stimulation	0.21	0.649	0.005	**6.78**	**0.013**	**0.139**
	Dimension	0.02	0.889	< 0.001	**10.52**	**0.002**	**0.200**
	Age × stimulation	**4.18**	**0.047**	**0.090**	0.04	0.851	0.001
	Age × dimension	0.01	0.921	< 0.001	2.23	0.143	0.050
	Stimulation × dimension	0.24	0.627	0.006	1.79	0.188	0.041
	Age × stimulation × dimension	0.67	0.418	0.016	< 0.01	0.996	< 0.001
P2	Age	**6.89**	**0.012**	**0.141**	3.07	0.087	0.068
	Stimulation	**33.59**	**< 0.001**	**0.444**	2.50	0.121	0.056
	Dimension	**5.62**	**0.022**	**0.118**	2.96	0.093	0.066
	Age × stimulation	0.02	0.875	0.001	2.75	0.105	0.061
	Age × dimension	0.47	0.495	0.011	0.08	0.775	0.002
	Stimulation × dimension	2.66	0.111	0.059	0.52	0.474	0.012
	Age × stimulation × dimension	**8.84**	**0.005**	**0.174**	0.02	0.890	< 0.001
MMN	Age	0.42	0.521	0.010	0.82	0.371	0.019
	Stimulation	0.19	0.664	0.005	1.65	0.207	0.038
	Dimension	**48.99**	**< 0.001**	**0.538**	0.39	0.536	0.009
	Age × stimulation	0.15	0.700	0.004	0.14	0.710	0.003
	Age × dimension	**7.50**	**0.009**	**0.151**	0.73	0.398	0.017
	Stimulation × dimension	**7.44**	**0.009**	**0.150**	0.43	0.517	0.010
	Age × stimulation × dimension	0.01	0.919	< 0.001	0.43	0.515	0.010
P3a	Age	3.68	0.062	0.081	0.09	0.761	0.002
	Stimulation	1.10	0.300	0.026	**16.20**	**< 0.001**	**0.278**
	Dimension	**23.24**	**< 0.001**	**0.356**	**7.42**	**0.009**	**0.150**
	Age × stimulation	0.26	0.610	0.006	0.76	0.389	0.018
	Age × dimension	0.84	0.364	0.020	**4.68**	**0.036**	**0.100**
	Stimulation × dimension	0.22	0.639	0.005	**9.05**	**0.004**	**0.177**
	Age × stimulation × dimension	< 0.01	0.959	< 0.001	0.71	0.404	0.017
P3b	Age	< 0.01	0.969	< 0.001	0.17	0.684	0.004
	Stimulation	**10.40**	**0.002**	**0.198**	2.30	0.137	0.052
	Dimension	**5.34**	**0.026**	**0.113**	**139.69**	**< 0.001**	**0.769**
	Age × stimulation	0.26	0.616	0.006	0.54	0.466	0.013
	Age × dimension	1.41	0.242	0.032	**4.82**	**0.034**	**0.103**
	Stimulation × dimension	**15.28**	**< 0.001**	**0.267**	**29.91**	**< 0.001**	**0.416**
	Age × stimulation × dimension	1.35	0.252	0.031	0.43	0.516	0.010

Significant results are printed in bold.

**TABLE 3:** Peak amplitudes and peak latencies of P1, N1, and P2 as a function of age (younger, older), stimulation (auditory-only, audio-visual), and target dimension (azimuth, distance).

	P1		N1		P2	
	Amplitude (μV)	Latency (ms)	Amplitude (μV)	Latency (ms)	Amplitude (μV)	Latency (ms)
Factor level/combination	*M (SD)*	*M (SD)*	*M (SD)*	*M (SD)*	*M (SD)*	*M (SD)*
Younger	0.89 (0.16)	61.43 (5.26)	−0.44 (0.16)	92.35 (3.18)	2.27 (1.01)	195.08 (29.84)
Older	1.32 (0.07)	52.65 (5.44)	−1.20 (0.12)	97.34 (5.20)	3.94 (0.83)	222.97 (18.87)
Auditory-only	1.11 (0.26)	53.83 (7.27)	−0.85 (0.27)	92.73 (5.43)	2.30 (0.90)	196.41 (34.67)
Audio-visual	1.10 (0.24)	60.25 (4.75)	−0.79 (0.51)	96.97 (3.34)	3.91 (1.00)	221.64 (9.70)
Azimuth	1.16 (0.15)	53.81 (7.51)	−0.81 (0.40)	91.92 (4.08)	3.40 (1.26)	223.48 (22.75)
Distance	1.05 (0.31)	60.27 (4.33)	−0.83 (0.42)	97.77 (3.97)	2.82 (1.17)	194.57 (26.03)
Younger, auditory-only	0.87 (0.10)	57.95 (5.54)	−0.59 (0.06)	90.39 (3.45)	1.44 (0.09)	169.25 (19.01)
Younger, audio-visual	0.90 (0.20)	64.91 (0.80)	−0.29 (0.06)	94.32 (0.83)	3.10 (0.82)	220.91 (5.61)
Older, auditory-only	1.34 (0.09)	49.70 (6.44)	−1.11 (0.07)	95.07 (6.04)	3.15 (0.35)	223.57 (23.70)
Older, audio-visual	1.30 (0.04)	55.59 (0.76)	−1.30 (0.09)	99.61 (2.76)	4.73 (0.16)	222.36 (12.56)
Younger, azimuth	1.03 (0.08)	58.39 (5.97)	−0.43 (0.15)	90.77 (3.83)	2.65 (1.27)	207.11 (19.46)
Younger, distance	0.75 (0.06)	64,48 (1.19)	−0.44 (0.17)	93.93 (0.85)	1.90 (0.43)	183.05 (32.91)
Older, azimuth	1.29 (0.05)	49.23 (5.96)	−1.19 (0.08)	93.07 (4.04)	4.15 (0.68)	239.84 (10.99)
Older, distance	1.35 (0.08)	56.07 (0.59)	−1.21 (0.16)	101.91 (0.97)	3.73 (0.93)	206.09 (4.08)
Auditory-only, azimuth	1.12 (0.16)	47.93 (4.66)	−0.86 (0.30)	88.14 (1.46)	2.44 (1.06)	216.45 (30.46)
Auditory-only, distance	1.10 (0.32)	59.73 (3.76)	−0.77 (0.48)	97.32 (3.78)	2.16 (0.68)	176.36 (26.13)
Audio-visual, azimuth	1.20 (0.12)	59.68 (4.67)	−0.83 (0.24)	95.70 (1.50)	4.35 (0.48)	230.50 (4.37)
Audio-visual, distance	1.00 (0.29)	60.82 (4.81)	−0.82 (0.55)	98.23 (4.14)	3.48 (1.19)	212.77 (3.23)
Younger, auditory-only, azimuth	0.96 (0.04)	52.50 (0.51)	−0.57 (0.06)	87.14 (1.36)	1.40 (0.09)	187.95 (2.44)
Younger, auditory-only, distance	0.78 (0.04)	63.41 (0.50)	−0.60 (0.05)	93.64 (0.58)	1.49 (0.06)	150.55 (0.91)
Younger, audio-visual, azimuth	1.09 (0.04)	64.27 (0.46)	−0.30 (0.06)	94.41 (0.67)	3.90 (0.16)	226.27 (0.63)
Younger, audio-visual, distance	0.71 (0.05)	65.55 (0.51)	−0.27 (0.05)	94.23 (0.97)	2.31 (0.14)	215.55 (1.92)
Older, auditory-only, azimuth	1.27 (0.05)	43.36 (0.66)	−1.15 (0.07)	89.14 (0.64)	3.48 (0.12)	244.95 (13.83)
Older, auditory-only, distance	1.42 (0.06)	56.05 (0.58)	−1.07 (0.05)	101.00 (0.76)	2.83 (0.09)	202.18 (0.66)
Older, audio-visual, azimuth	1.31 (0.04)	55.09 (0.53)	−1.23 (0.06)	97.00 (0.82)	4.81 (0.13)	234.73 (1.12)
Older, audio-visual, distance	1.28 (0.04)	56.09 (0.61)	−1.36 (0.05)	102.23 (0.75)	4.64 (0.13)	210.00 (1.27)

**TABLE 4:** Mean amplitudes and fractional area latencies (FAL) of MMN, P3a, and P3b as a function of age (younger, older), stimulation (auditory-only, audio-visual), and target dimension (azimuth, distance).

	MMN		P3a		P3b	
	Amplitude (μV)	Latency (ms)	Amplitude (μV)	Latency (ms)	Amplitude (μV)	Latency (ms)
Factor level/combination	*M (SD)*	*M (SD)*	*M (SD)*	*M (SD)*	*M (SD)*	*M (SD)*
Younger	−0.90 (0.71)	156.66 (21.25)	0.68 (0.53)	284.36 (49.30)	2.78 (0.45)	485.18 (58.61)
Older	−0.74 (0.36)	132.95 (26.38)	1.63 (0.34)	290.66 (24.94)	2.81 (0.43)	473.16 (39.09)
Auditory-only	−0.85 (0.38)	130.78 (9.44)	1.02 (0.67)	314.68 (37.71)	3.08 (0.42)	493.49 (63.45)
Audio-visual	−0.78 (0.71)	158.83 (30.74)	1.29 (0.61)	260.34 (12.53)	2.51 (0.23)	464.85 (24.42)
Azimuth	−1.30 (0.32)	153.09 (21.52)	1.55 (0.45)	271.59 (19.30)	2.94 (0.56)	437.50 (8.88)
Distance	−0.33 (0.26)	136.52 (28.81)	0.76 (0.58)	303.43 (46.77)	2.65 (0.18)	520.84 (38.01)
Younger, auditory-only	−0.97 (0.50)	138.55 (5.00)	0.47 (0.45)	317.41 (51.15)	3.11 (0.42)	506.45 (71.77)
Younger, audio-visual	−0.83 (0.87)	174.77 (14.75)	0.88 (0.53)	251.32 (8.18)	2.45 (0.12)	463.91 (29.56)
Older, auditory-only	−0.74 (0.10)	123.02 (5.67)	1.56 (0.30)	311.95 (15.69)	3.05 (0.42)	480.52 (51.47)
Older, audio-visual	−0.73 (0.50)	142.89 (34.26)	1.70 (0.36)	269.36 (9.18)	2.57 (0.29)	465.80 (18.18)
Younger, azimuth	−1.58 (0.14)	153.61 (18.30)	1.15 (0.27)	255.80 (12.23)	3.01 (0.53)	504.72 (76.48)
Younger, distance	−0.22 (0.27)	159.70 (23.67)	0.21 (0.21)	312.93 (55.66)	2.56 (0.17)	482.96 (58.12)
Older, azimuth	−1.03 (0.20)	152.57 (24.52)	1.95 (0.13)	287.39 (9.65)	2.88 (0.59)	459.22 (46.55)
Older, distance	−0.44 (0.21)	113.34 (4.42)	1.31 (0.07)	293.93 (33.81)	2.73 (0.14)	468.22 (41.56)
Auditory-only, azimuth	−1.15 (0.32)	131.98 (3.94)	1.38 (0.49)	282.02 (15.11)	3.49 (0.10)	432.66 (4.61)
Auditory-only, distance	−0.56 (0.10)	129.59 (12.73)	0.66 (0.63)	347.34 (21.59)	2.67 (0.09)	554.32 (23.46)
Audio-visual, azimuth	−1.46 (0.25)	174.20 (2.98)	1.72 (0.35)	261.16 (17.38)	2.40 (0.15)	442.34 (9.51)
Audio-visual, distance	−0.10 (0.14)	143.45 (37.67)	0.87 (0.51)	259.52 (3.76)	2.63 (0.24)	487.36 (8.87)
Younger, auditory-only, azimuth	−1.46 (0.05)	135.55 (0.86)	0.91 (0.09)	267.41 (3.76)	3.52 (0.10)	435.59 (3.94)
Younger, auditory-only, distance	−0.48 (0.06)	141.55 (5.62)	0.04 (0.10)	367.41 (10.22)	2.71 (0.07)	577.32 (3.11)
Younger, audio-visual, azimuth	−1.69 (0.10)	171.68 (1.04)	1.38 (0.13)	244.18 (3.08)	2.49 (0.12)	435.95 (8.47)
Younger, audio-visual, distance	0.03 (0.06)	177.86 (20.60)	0.38 (0.13)	258.45 (4.55)	2.42 (0.10)	491.86 (8.97)
Older, auditory-only, azimuth	−0.84 (0.04)	128.41 (2.09)	1.85 (0.07)	296.64 (2.48)	3.46 (0.09)	429.73 (3.17)
Older, auditory-only, distance	−0.64 (0.03)	117.64 (0.85)	1.27 (0.06)	327.27 (2.47)	2.64 (0.09)	531.32 (3.06)
Older, audio-visual, azimuth	−1.23 (0.06)	176.73 (1.93)	2.05 (0.09)	278.14 (2.32)	2.30 (0.10)	448.73 (5.28)
Older, audio-visual, distance	−0.24 (0.02)	109.05 (0.84)	1.36 (0.05)	260.59 (2.42)	2.83 (0.11)	482.86 (6.19)

#### 3.2.1 P1-N1-P2

The P1 amplitude was descriptively greater in older than in younger participants. In addition, there was an interaction between age and target dimension. Irrespective of the modality of stimulation, older participants showed numerically higher P1 amplitudes than younger participants, particularly for distance targets. However, this difference narrowly missed statistical significance (*M*_*diff*_ = 0.60, *t* = 2.23, *p* = 0.063). The age difference for azimuth targets was also not significant (*M*_*diff*_ = 0.26, *t* = 1.04, *p* = 0.307). In addition, there was an interaction between stimulation and target dimension for P1 latency. *Post-hoc* tests showed that under auditory-only stimulation, P1 latency was significantly shorter for azimuth than for distance targets (*M* = −11.80 ms, *t* = −6.80, *p* < 0.001). In contrast, under audio-visual stimulation, the latency difference between azimuth and distance targets was not significant (*M*_*diff*_ = −1.14 ms, *t* = −0.66, *p* = 0.512). This pattern indicates that the presence of visual information reduced the P1 latency difference between azimuth and distance targets.

The N1 was greater in older than in younger participants. In addition, there was an interaction between age and stimulation. *Post-hoc* tests, however, did not reveal significant stimulation effects within each age group. Younger participants showed a non-significant reduction in N1 negativity under audio-visual compared with auditory-only stimulation (*M*_*diff*_ = 0.30, *t* = 1.87, *p* = 0.150), whereas older participants showed a non-significant effect in the opposite direction (*M*_*diff*_ = −0.19, *t* = −1.06, *p* = 0.299). The N1 latency was shorter with azimuth, than distance targets and with auditory-only, than with audio-visual stimulation.

Overall, the P2 was greater in older than in younger participants, with audio-visual stimulation than with auditory-only stimulation, and with azimuth than with distance targets. In addition, there was a three-way interaction between age, stimulation, and dimension. *Post-hoc* analyses revealed that in younger participants, P2 amplitudes were strongly enhanced by audio-visual stimulation for azimuth targets (*M*_*diff*_ = 2.50, *t* = 4.47, *p* < 0.001), whereas this enhancement was smaller and not statistically significant for distance targets (*M*_*diff*_ = 0.82, *t* = 1.42, *p* = 0.172). In older participants, P2 amplitudes increased with audio-visual stimulation for both azimuth (*M*_*diff*_ = 1.33, *t* = 3.58, *p* = 0.002) and distance targets (*M*_*diff*_ = 1.82, *t* = 5.71, *p* < 0.001). Additional age-group comparisons showed that older participants had larger P2 amplitudes than younger participants under auditory-only stimulation for both azimuth (*M*_*diff*_ = 2.08, *t* = 3.03, *p* = 0.012) and distance targets (*M*_*diff*_ = 1.34, *t* = 2.89, *p* = 0.012). Under audio-visual stimulation, older participants showed larger P2 amplitudes for distance targets (*M*_*diff*_ = 2.33, *t* = 2.75, *p* = 0.012), but not for azimuth targets (*M*_*diff*_ = 0.91, *t* = 0.97, *p* = 0.338). No effects were found for P2 latency.

#### 3.2.2 Mismatch negativity

Figure 5 shows the grand-averaged ERP waveforms, including the P1, N1, and P2 components, across the different target dimensions, age groups, and stimulation modalities. The MMN amplitude was greater with azimuth than with distance targets. This effect of dimension was modulated by stimulation: Under auditory-only stimulation, MMN amplitudes were significantly more negative for azimuth than for distance targets (*M*_*diff*_ = −0.59, *t* = −3.79, *p* < 0.001). Under audio-visual stimulation, this azimuth-distance difference was also significant and more pronounced (*M*_*diff*_ = −1.36, *t* = −5.85, *p* < 0.001). Thus, the azimuth-related MMN effect was present in both stimulation conditions but was amplified by the presence of visual cues. Furthermore, there was an interaction between dimension and age: MMN amplitudes were significantly more negative for azimuth than for distance targets in both younger participants (*M*_*diff*_ = −1.35, *t* = −6.60, *p* < 0.001) and older participants (*M*_*diff*_ = −0.59, *t* = −3.15, *p* = 0.005). These results indicate that the effect of spatial dimension on MMN amplitude was age-dependent. Specifically, the azimuth-distance difference was attenuated in older participants, suggesting a reduced differentiation between azimuth and distance deviations with age. No effects were found for the MMN latency.

#### 3.2.3 P3a

The P3a amplitude was greater with azimuth, than distance targets, and descriptively higher in older than younger participants. The P3a latency was increased for auditory-only compared with audio-visual targets, and for distance compared with azimuth targets.

The P3a latency was increased for auditory-only distance targets, according to an interaction of dimension and stimulation. Furthermore, there was an interaction between dimension and age. *Post-hoc* comparisons showed that older participants had numerically longer P3a latencies than younger participants for azimuth targets, but this difference did not reach significance (*M*_*diff*_ = 31.59 ms, *t* = 1.88, *p* = 0.135). Further, no significant age difference was observed for distance targets (*M*_*diff*_ = −19.00 ms, *t* = −0.66, *p* = 0.516). An additional significant interaction between stimulation and dimension showed that under auditory-only stimulation, P3a latency was significantly shorter for azimuth than for distance targets (*M*_*diff*_ = −65.32 ms, *t* = −3.04, *p* = 0.008). Under audio-visual stimulation, no significant latency difference between azimuth and distance targets was observed (*M*_*diff*_ = 1.64 ms, *t* = 0.21, *p* = 0.833). This pattern suggests that audio-visual cues reduced the latency difference between spatial dimensions.

#### 3.2.4 P3b

The P3b was greater with auditory-only than with audio-visual stimulation, and with azimuth than with distance targets. This main effect was modulated by an interaction between stimulation and target dimension. Specifically, in the auditory-only condition, P3b amplitude was significantly larger for azimuth than for distance targets (*M*_*diff*_ = 0.82, *t* = 4.35, *p* < 0.001). In contrast, in the audio-visual condition, P3b amplitude did not differ significantly between azimuth and distance targets (*M*_*diff*_ = −0.23, *t* = −1.26, *p* = 0.214). These results indicate that the largest P3b amplitude occurred for auditory-only azimuth targets, whereas audio-visual stimulation largely reduced the difference between spatial dimensions. The P3b area latency was larger for distance than for azimuth targets. This dimension effect was modulated by stimulation: Although the azimuth-distance difference was significant in both stimulation conditions, it was larger under auditory-only stimulation (*M*_*diff*_ = −121.66 ms, *t* = −14.15, *p* < 0.001) than under audio-visual stimulation (*M*_*diff*_ = −45.02 ms, *t* = −4.05, *p* < 0.001). Furthermore, when examining age groups separately, P3b latencies were significantly shorter for azimuth than for distance targets in both younger participants (*M*_*diff*_ = −98.82 ms, *t* = −9.52, *p* < 0.001) and older participants (*M*_*diff*_ = −67.86 ms, *t* = −7.10, *p* < 0.001). Thus, the latency difference between spatial dimensions was present in both age groups, although it was numerically larger in younger than in older participants.

**FIGURE 6:**
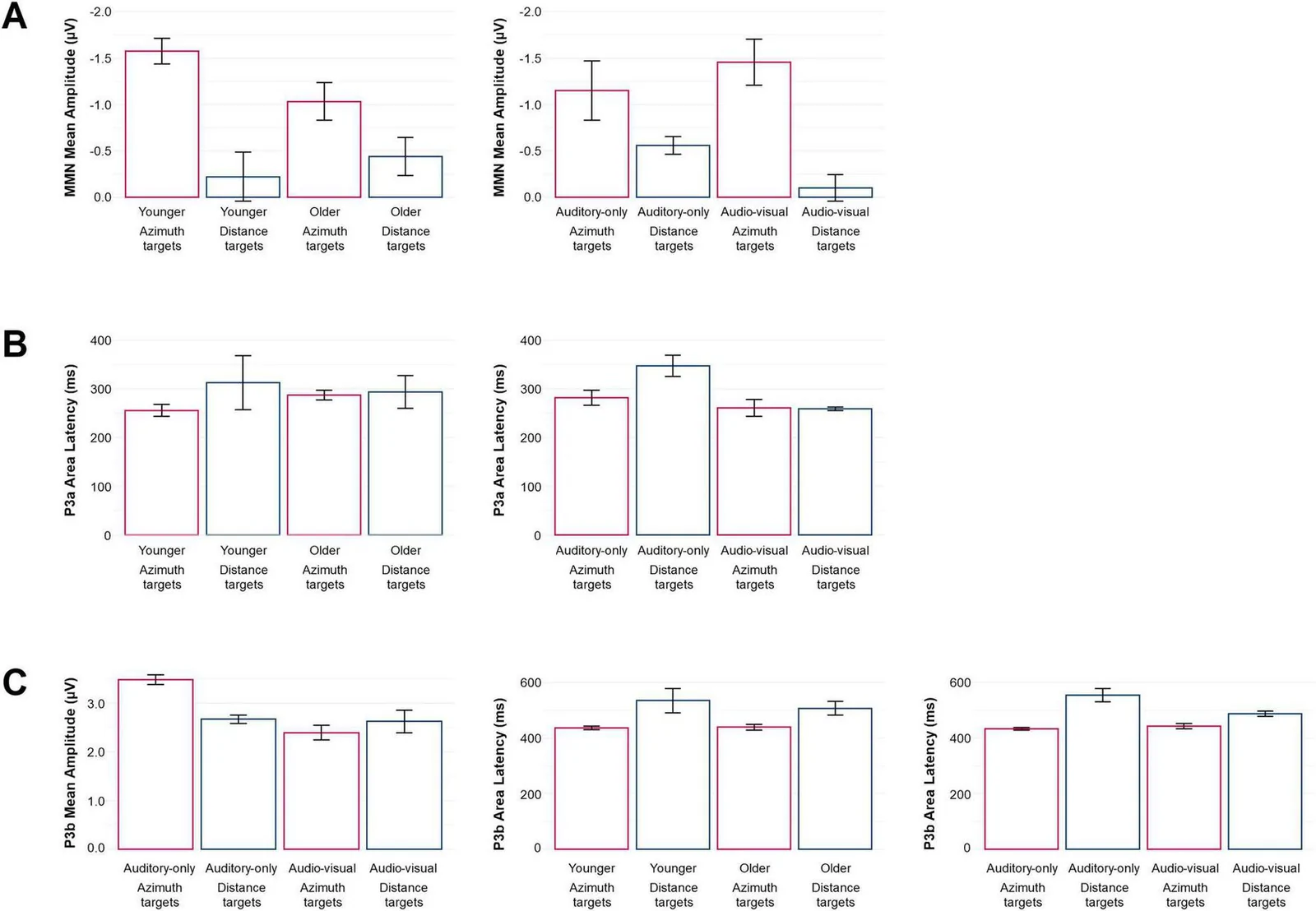
Visualization of significant interaction effects for the amplitudes and latencies of **(A)** MMN, **(B)** P3a, and **(C)** P3b components.

Figure 6 further visualizes the significant interaction effects for the amplitudes and latencies of MMN, P3a, and P3b.

## 4 Discussion

The present study investigated whether spatial change detection in azimuth and distance benefits from audio-visual perception over purely auditory processing, with a specific focus on possible differences related to age. We therefore extended a previously introduced auditory spatial oddball paradigm to include congruent audio-visual stimuli, allowing a direct contrast between unimodal auditory and bimodal audio-visual change detection.

### 4.1 Behavioral performance: audio-visual benefits but preserved performance in older adults

Our behavioral results revealed a clear dissociation between detecting changes in azimuth versus distance. Across both age groups, participants demonstrated significantly higher accuracy and faster responses for spatial changes in the horizontal plane compared to depth. This finding aligns with fundamental principles of auditory spatial perception: sound localization in azimuth relies on salient binaural cues (ITD, ILD), whereas distance perception depends on more subtle monaural cues including intensity variations, spectral characteristics, and the direct-to-reverberant energy ratio, which are often more difficult to detect reliably, especially in reverberant environments (Arend et al., 2021; Kolarik et al., 2016; Zahorik et al., 2005), thereby likely contributing to differences in perceptual salience and discriminability between azimuth and distance targets.

Spatially congruent visual stimuli substantially improved detection performance, particularly for distance changes. In the audio-visual condition, performance differences between azimuth and distance detection were eliminated, suggesting that visual information can mitigate limitations associated with auditory distance cues. This pattern is broadly consistent with principles of multisensory integration, whereby the brain combines information from different modalities to reduce perceptual uncertainty (Pichora-Fuller, 2008; Teder-Sälejärvi et al., 2005). Visual depth cues provide robust spatial information that can enhance or dominate auditory perception when both modalities are available (Anderson and Zahorik, 2014; Calcagno et al., 2012; Zahorik and Wightman, 2001).

Contrary to our hypothesis, older adults did not benefit more from audio-visual stimulation than younger adults. Despite well-documented age-related declines in both auditory and visual processing (e.g., Xiao et al., 2021), older participants demonstrated comparable detection accuracy and response times across both conditions. This suggests that multisensory integration mechanisms remain effective in aging, although the absence of larger behavioral benefits may also be influenced by differences in perceptual discriminability between azimuth and distance changes in the auditory-only condition. Extending previous work on purely auditory spatial change detection (Stodt et al., 2025), this pattern suggests that older adults may employ compensatory cognitive strategies that maintain functional performance. In line with this, studies using response time paradigms have shown that multisensory stimulation can facilitate performance in both younger and older adults, with comparable or even enhanced benefits for older adults depending on the stimulus combination (Mahoney et al., 2011). This suggests that multisensory integration mechanisms remain effective in aging, even if they do not always lead to larger behavioral advantages. This finding is consistent with Xiong et al. (2022) who demonstrated that although older adults show declines in auditory and visual localization when tested separately, the interactions between these modalities remain largely intact. Our results extend this to spatial change detection, indicating that older adults retain the ability to leverage multimodal information for detecting spatial changes in the environment. However, the relatively high accuracy levels, particularly in the audio-visual condition, may have resulted in ceiling effects that limited our ability to detect subtle age-related differences. This consideration is important when interpreting the absence of significant age effects.

### 4.2 Neural correlates: age-related differences in spatial change processing

While behavioral performance was similar across age groups, electrophysiological data revealed substantial age-related differences in underlying neural mechanisms. These findings demonstrate that equivalent behavioral outcomes can be achieved through distinct patterns of neural processing, consistent with theories emphasizing neural compensation in cognitive aging (Cabeza et al., 2002; Reuter-Lorenz and Cappell, 2008).

#### 4.2.1 Early sensory processing: enhanced attention allocation in older adults

The P1-N1-P2 complex revealed pronounced age effects. Both N1 and P2 showed significantly larger amplitudes in older compared to younger adults, while P1 amplitudes were descriptively enhanced in older adults.

The N1, which marks attentional orienting to novel stimuli (Näätänen and Picton, 1987), consistently increases with age across auditory paradigms (Alain and Woods, 1999; Amenedo and Díaz, 1998; Tomé et al., 2015). This enhancement is typically interpreted as a compensatory mechanism, whereby older adults allocate greater attentional resources to compensate for declines in peripheral auditory function and automatic processing efficiency (Finnigan et al., 2011; Strömmer et al., 2017). The enhanced N1 in older compared to younger adults is broadly consistent with this interpretation. However, the interaction between age and stimulation should be interpreted cautiously. Although younger participants showed a numerical reduction in N1 negativity under audio-visual compared with auditory-only stimulation, and older participants showed a numerical effect in the opposite direction, neither simple effect reached significance. Thus, the N1 results provide evidence for enhanced early sensory responses in older adults, but only limited evidence for reliable age-dependent modulation of N1 amplitudes by audio-visual stimulation. Furthermore, N1 latencies were shorter for azimuth than distance targets and for auditory-only compared with audio-visual stimulation, suggesting that early sensory encoding is faster for more salient or simpler spatial cues (Kean and Lambert, 2003), which may partly reflect differences in perceptual discriminability between azimuth and distance stimuli. The slightly prolonged N1 in the audio-visual condition may reflect the additional processing demands of integrating information across modalities, even as this integration may be associated with amplified neural responses. Notably, previous studies on speech perception have reported shorter N1 latencies with multisensory stimulation (Alsius et al., 2014; Winneke and Phillips, 2011), suggesting that the effects of cross-modal integration on early sensory processing may vary depending on task or stimulus characteristics.

Similarly, audio-visual stimulation enhanced P2 amplitudes, but this effect depended on both age group and spatial dimension. In younger participants, P2 amplitudes were higher for audio-visual than auditory-only stimulation for azimuth targets, whereas no significant audio-visual enhancement was observed for distance targets. In older participants, audio-visual stimulation enhanced P2 amplitudes for both azimuth and distance targets. This suggests that younger adults showed a more dimension-specific audio-visual benefit, primarily for azimuth cues, whereas older adults showed a broader audio-visual enhancement across spatial dimensions. The P2 is associated with stimulus classification and relevance evaluation (Arnott et al., 2011) and its enhancement may therefore reflect increased neural resources allocated to processing and integrating multisensory spatial information. Additional age-group comparisons further indicated that older adults showed larger P2 amplitudes than younger adults under auditory-only stimulation for both azimuth and distance targets, as well as under audio-visual stimulation for distance targets. In contrast, the age difference under audio-visual stimulation for azimuth targets was not significant. Thus, the significant main effect of age on P2 amplitude appears to reflect robust age-related enhancement in several, but not all, stimulation-by-dimension combinations. Specifically, younger adults may have benefited from visual information particularly when processing azimuth cues, whereas older adults may have recruited additional resources especially when visual information supported the processing of distance cues, which are typically less salient and more challenging to evaluate.

Even the earliest component, the P1, showed indications of age-related modulation rather than robust age-related differences for distance targets. Older adults displayed numerically higher P1 amplitudes than younger adults, particularly for distance targets, although this difference narrowly missed significance. This pattern may suggest that compensatory mechanisms begin to emerge already at early stages of sensory encoding, especially for less salient spatial cues such as distance (Eggermont and Ponton, 2002; Grunwald et al., 2003). Furthermore, the P1 latency was also modulated by spatial dimension and stimulation modality: Under auditory-only stimulation, P1 latency was significantly shorter for azimuth than for distance targets, whereas this latency difference was not significant under audio-visual stimulation. This indicates that visual information reduced the latency difference between spatial dimensions, resulting in more similar processing times for azimuth and distance cues. While direct evidence for the disappearance of spatial-latency differences in audio-visual conditions is limited, previous ERP research has shown very early audio-visual integration and attentional modulation of early components (e.g., Talsma et al., 2009), suggesting that multisensory input can indeed influence early sensory timing.

#### 4.2.2 Automatic change detection: reduced mismatch negativity in older adults

The MMN, indexing automatic change detection (Escera and Corral, 2007; Näätänen et al., 2007; Rutiku et al., 2024), was more pronounced for azimuth than distance targets, reflecting the higher salience or discriminability of spatial changes in the horizontal plane. Importantly, this azimuth-related enhancement was present in both stimulation conditions, but was more pronounced under audio-visual stimulation. Thus, visual cues appeared to amplify, but not solely account for, the azimuth-related MMN effect. In addition, the MMN revealed an interaction between age and target dimension. Both younger and older adults showed more negative MMN amplitudes for azimuth than for distance targets, but this azimuth-distance difference was attenuated in older adults. This suggests that aging was associated with reduced differentiation between spatial dimensions during automatic change detection. This pattern is consistent with previous research showing age-related impairments in automatic auditory change detection and spatial deviance processing (Cheng et al., 2013; Cooper et al., 2006; Getzmann et al., 2024; Ruzzoli et al., 2012), including findings by Freigang et al., (2014), who demonstrated age-related impairments in automatic processing of spatial changes, particularly for peripheral locations. Within predictive coding frameworks, MMN represents lower-level prediction errors, that is, violations of the brain’s implicit model of sensory regularities in both auditory and visual domain (Stefanics et al., 2014; Wacongne et al., 2012; Winkler and Czigler, 2012). The reduced azimuth-distance differentiation in older adults may therefore reflect a degraded or less precise internal model of spatial regularities. Notably, age-related differences in MM amplitudes appeared most pronounced for azimuth targets. For distance targets, older participants showed slightly larger MMNs than younger participants, although responses were generally smaller than for azimuth targets, likely reflecting the lower salience of distance changes in line with behavioral findings.

It should be noted that, in the present paradigm, the observed MMN may not be entirely independent of early auditory components such as the N1 and acoustic change responses, due to an overlap of successive ERP components in oddball paradigms (Escera et al., 1998). Given the reported age-related differences in N1, early sensory processing and refractoriness may have contributed to the measured deviance-related activity. However, this does not alter the interpretation of the findings in terms of automatic change detection.

The P3a component, reflecting involuntary attention orienting toward unexpected stimuli (Sanada et al., 2025; Schubert et al., 2001), showed a similar pattern, with higher amplitudes and shorter latencies for azimuth than distance targets, consistent with previous findings demonstrating that auditory stimuli with greater perceptual distinctiveness elicit stronger and faster P3a responses (Comerchero and Polich, 1998). Although the interaction between age and dimension suggested age-related modulation of P3a latency, follow-up comparisons did not reveal significant age differences for either azimuth or distance targets. Older participants showed only numerically longer P3a latencies than younger participants for azimuth targets, whereas no age-related latency difference was observed for distance targets. Importantly, the latency difference between azimuth and distance targets was significant under auditory-only stimulation, but absent under audio-visual stimulation, suggesting that multisensory cues may reduce spatial-dimension-related differences in attentional orienting latency and support more consistent attentional orienting across spatial dimensions (Santangelo et al., 2008).

#### 4.2.3 Later evaluative processing: task difficulty moderates age effects

The P3b component, reflecting stimulus evaluation, context updating, and resource allocation (Kok, 2001; Polich, 2007; Verleger et al., 2005) showed effects of both target dimension and stimulation. The P3b amplitude was reduced and latency prolonged for distance compared to azimuth targets, particularly in the auditory-only condition. This suggests that the processing of distance changes in the auditory environment may require greater cognitive effort than azimuth changes (Stodt et al., 2025), potentially reflecting lower reliability and discriminability of distance cues, whereas this difference is reduced in the audio-visual condition. When examining age groups separately, P3b latencies were shorter for azimuth than for distance targets in both younger and older participants, although this latency difference was numerically larger in younger than in older participants. Thus, the P3b latency results do not indicate an age-related delay specifically for azimuth targets, but rather suggest that the temporal advantage for azimuth over distance processing was present in both age groups. This suggests that, when spatial cues are more difficult to evaluate, both age groups require extended processing time, reducing the emergence of clear age-specific latency differences (Katayama and Polich, 1998; Verleger et al., 2014).

Taken together, the ERP patterns reveal a nuanced picture: While behavioral performance was preserved, older adults showed altered automatic spatial processing, reflected in reduced MMN differentiation between azimuth and distance deviations, alongside compensatory enhancements in early sensory processing, as indicated by increased N1 and P2 amplitudes. This dissociation suggests that older adults maintain performance through engagement of additional neural resources, consistent with compensation accounts like the CRUNCH hypothesis (Reuter-Lorenz and Cappell, 2008).

### 4.3 Audio-visual integration across the lifespan

A central question was whether audio-visual stimulation would differentially benefit older adults. Our hypothesis assumed that sensory declines might increase reliance on multisensory integration. However, both age groups showed comparable benefits from audio-visual stimulation, with similar improvements relative to auditory-only conditions. In line with this, previous work has shown that multisensory stimulation can yield comparable or even enhanced response time benefits in older adults, particularly for certain stimulus combinations, suggesting that multisensory integration remains effective and may be flexibly upregulated in aging (Laurienti et al., 2006). This suggests that audio-visual integration mechanisms in spatial perception may be relatively preserved in healthy aging, at least for detecting spatially congruent changes in simple environments. This preservation contrasts with declines in many unisensory functions and may reflect the robustness of multisensory integration processes involving widespread cortical networks (as proposed by Xiong et al., 2022).

In particular, the enhanced early ERPs in older adults, especially the overall N1 enhancement and the pronounced P2 effects, may represent the neural substrate of compensatory integration. While younger adults may integrate information relatively automatically, older adults may engage in more effortful processing to achieve similar outcomes, consistent with findings of increased neural activation in older adults achieving equivalent performance (Cabeza et al., 2002; Grady, 2012). In other words, for azimuth targets, visual information enhanced P2 amplitudes in both age groups and appeared to reduce age-related amplitude differences. For distance targets, audio-visual benefits appeared more pronounced in older adults (larger P2 amplitudes), suggesting particular benefit from visual information when auditory cues are less salient, quite in line with the principle of inverse effectiveness in multisensory integration (de Dieuleveult et al., 2017; Schneeberger et al., 2024; Zou et al., 2017).

Our findings extend previous work examining auditory spatial change detection in real versus virtual environments (Stodt et al., 2024, 2025). We previously demonstrated that virtual auditory environments could reliably replicate neural and behavioral signatures of auditory spatial processing, though some differences emerged in MMN and P3b. The current study builds on this by adding the visual modality. Consistent with our auditory-only findings, we again observed no age-related differences in detection accuracy, suggesting robustness across unimodal and multimodal formats. The reduced MMN differentiation between azimuth and distance deviations and enhanced N1/P2 in older adults also replicate earlier findings, strengthening confidence in these as reliable markers of age-related changes in spatial auditory processing. However, the current study also showed that while distance changes were processed differently than azimuth changes in auditory modality, visual information modulated these dimension-related differences. Specifically, visual cues reduced several azimuth-distance differences in later processing and latency measures, while the MMN azimuth-distance difference was amplified under audio-visual stimulation. This suggests that visual information does not uniformly reduce spatial processing differences, but can differentially shape neural processing depending on the processing stage and ERP component. This highlights the importance of considering multimodal contexts in spatial cognition research. The broader literature on spatial change detection has yielded mixed findings regarding age effects (Klencklen et al., 2012). Some studies report age-related declines in visual change detection (Costello et al., 2010; Tran et al., 2021), while others find preserved performance when adequate processing time is provided. Our findings contribute by demonstrating that behavioral performance can be preserved despite substantial neural changes. Studies reporting declines may have exceeded compensatory capacity, whereas those finding preserved performance (like ours) may have used parameters allowing effective compensation.

### 4.4 Implications and limitations

From a theoretical point of view, our findings contribute to several aspects in cognitive neuroscience of aging. First, they support compensation theories, demonstrating that preserved behavioral performance can coexist with substantially altered neural processing. The increased early sensory ERPs coupled with reduced MMN differentiation between azimuth and distance deviations suggest that successful cognitive aging involves a shift toward more resource-intensive, attention-demanding strategies. Second, our results show that preserved audio-visual integration benefits can occur despite age-related changes in unisensory processing, suggesting that multisensory mechanisms may be either relatively age-invariant or subject to compensatory enhancement. This has implications for predictive coding accounts, suggesting that while the precision of spatial predictions or the differentiation of spatial regularities may decline, cross-modal integration capacity may remain largely intact. Finally, the pronounced differences between azimuth and distance processing across multiple levels demonstrate the importance of considering specific spatial dimensions and cue types in auditory perception. Models should account for differential salience and neural requirements of horizontal versus depth-based spatial changes, particularly in aging.

From a practical point of view, the apparent preservation of audio-visual spatial change detection in older adults has implications for everyday life activities: For example, audio-visual warning systems in vehicles or navigation aids could support multisensory integration to enhance older adults’ ability to detect spatial changes. Given that distance-related processing appeared to benefit from visual support at several processing stages, depth-based warnings (e.g., approaching obstacles) should incorporate visual elements. The same is true for future assistive technologies: Devices for older adults with sensory impairments could use the preserved multisensory integration, with hearing aids or cochlear implants being enhanced by incorporating visual feedback or augmented reality elements providing spatial information.

However, there is an important caveat: While behavioral performance was preserved, increased neural effort (indicated by enhanced early ERPs) suggests that maintaining performance may be more cognitively demanding for older individuals. In complex real-world environments with multiple concurrent demands, this increased load might result in fatigue and, consequently, potentially lead to performance decrements that were not observed in our relatively simple task. Future research should examine audio-visual spatial processing under divided attention or concurrent cognitive load.

In addition to that, there are several limitations to be acknowledged. First, azimuth and distance changes were not matched in terms of perceptual discriminability in the auditory-only condition. Distance cues are generally less precise and more context-dependent than azimuth cues, which likely made distance targets more difficult to detect. Consequently, the larger improvement observed in the audio-visual condition for distance may partly reflect differences in initial task performance rather than a dimension-specific benefit of multisensory integration. Without explicit matching of task difficulty or performance normalization, it remains difficult to fully disentangle effects of multisensory integration from differences in perceptual discriminability. Second, even though we applied a log-transformation to reduce skewness and mitigate potential ceiling effects, the high accuracy levels—particularly in the audio-visual condition—may still have limited our ability to detect subtle age-related differences. Future studies might therefore employ more challenging stimulus configurations. Third, and in line with that, our study used optimal listening and viewing conditions with minimal background noise and clearly visible stimuli. Real-world environments are typically more complex, with competing information, divided attention, and varied conditions. Whether our findings translate to such naturalistic settings remains to be established and requires extending to more complex, dynamic multi-source auditory environments (Russell, 2022). Virtual reality methodology offers promising approaches for increasing ecological validity while maintaining control (Parsons, 2015; Vasser and Aru, 2020). Fourth, although our older sample was carefully screened, it likely reflects a relatively healthy and high-functioning segment of the aging population. Thus, preserved multisensory integration might not extend to populations with greater sensory or cognitive challenges. In particular, it should be noted that the older participant group in the present study was relatively young in terms of cognitive aging. Therefore, it remains an open question whether the observed effects would generalize to later stages of aging, when age-related cognitive decline becomes more pronounced. Future studies including older and more heterogeneous samples are needed to determine whether multisensory integration and compensatory neural mechanisms remain stable or begin to decline in more advanced aging. Fifth, we examined spatial processing with participants maintaining fixed head and body position. Natural spatial perception typically involves head movements providing dynamic cues that substantially enhance localization and externalization (Brimijoin et al., 2013). Future studies incorporating head-tracking could investigate how dynamic spatial cues interact with multisensory integration and aging. Sixth, while hearing status was assessed via audiometry, vision status was based on self-report. Given the simplicity of the visual stimulus, which did not require detailed visual discrimination, any potential variability in visual acuity is expected to have only a limited impact on performance. In addition, although hearing thresholds were within the acceptable range, older adults showed significantly higher thresholds than younger adults. Thus, age-related effects may, at least in part, be influenced by differences in peripheral auditory encoding as well as central cortical processing, which should be considered when interpreting the observed group differences. Finally, the MMN was derived from an active oddball paradigm, in which participants were instructed to attend to spatial deviants. This differs from most of the classical MMN paradigms, where participants are typically instructed to ignore the auditory stimulation or attend to an unrelated task. Consequently, the deviance-related negativity observed here may reflect automatic change detection mechanisms, but it may also be modulated by attentional allocation and task relevance. This difference should be taken into account when relating the present findings to passive oddball studies.

## 5 Conclusion

This study demonstrates that audio-visual integration modulates neural processing of spatial change detection, especially for distance changes that are difficult to perceive in the auditory modality. Older adults exhibited comparable behavioral performance and evidence of preserved multisensory integration benefits despite age-related neural differences. Specifically, while early sensory processing was enhanced in older adults, automatic change detection showed reduced differentiation between azimuth and distance deviations, consistent with compensatory mechanisms that involve greater attentional resource allocation. Later evaluative processing was primarily modulated by spatial dimension and stimulation modality rather than by robust age-related delays. These findings support models of cognitive aging that emphasize neural compensation over the simple preservation of youthful processing. They also highlight the robustness of multisensory integration across the lifespan, underscoring the potential of multisensory environments to support spatial cognition in aging populations. Although older adults showed no behavioral deficits, the increased neural effort indicated by ERPs suggests that preserved performance may come at a cognitive cost. Future research should explore the limits of compensatory processing, especially in more complex or naturalistic settings. Nevertheless, these results offer evidence that older adults retain the ability to detect and respond to spatial changes, particularly when multisensory information is available.

## Data Availability

The raw data supporting the conclusions of this article will be made available by the authors, without undue reservation.
